# P160: The enablers and barriers to introducing "bare below the elbows" for hand hygiene behaviors: an exploratory study

**DOI:** 10.1186/2047-2994-2-S1-P160

**Published:** 2013-06-20

**Authors:** K McKay, R Shaban, E Coyne

**Affiliations:** 1 Infection Prevention & Control, Eastern Health, Box Hill, Brisbane, Australia; 2 Griffith Health Institute, Brisbane, Australia; 3 School of Nursing, Griffith University, Brisbane, Australia

## Introduction

Introduction of the ’bare below the elbows’ (BBE) guidelines within the National Health Service in the United Kingdom was met with complaints from many health care workers (HCW). BBE has been introduced in several states of Australia, and is expected to become mandatory in Victoria. There is no research indentifying the barriers and enablers to BBE nor any publicly available information on successful implementation programs. This study examines the barriers and enablers to the introduction of BBE within the 3 largest hospitals of the Eastern Health (EH) Network in outer Melbourne, Australia.

## Objectives

The aims of this study were to determine the extent to which the dress and adornment behaviours of HCW at EH were already consistent with the principles of BBE, and to determine the enablers and barriers to the introduction of BBE within clinical areas at EH.

## Methods

The study was descriptive and utilized both quantitative and qualitative methodology. Data was collected at the 3 major EH sites in 2 phases, a point prevalence audit which described the current hand adornment and HCW dress behaviours as compared to the BBE framework and focus groups to explore HCW opinions and feelings surrounding the changes inherent in BBE.

## Results

The audit showed that overall 11.7% of staff were compliant with BBE. Data was also examined according to site, gender, ward type, HCW group and BBE element. A picture of the dress and hand adornment practices of the target staff was thus able to be quantified. Barriers and enablers include the lack of a uniform, heating, clocks, pass holders and storage. In addition broader issues such as consequences, feedback, evidence, equity, identity, role modelling and organizational support were also identified.

## Conclusion

Achieving compliance with BBE is possible but would require consideration of multiple factors such as those illuminated by this work; medical staff may prove a difficult challenge with regard to sleeve length and the wearing of ties, while the removal of rings is an emotive area. It is anticipated that this data will allow the formulation of strategies to introduce these practice changes in an efficient, cost effective and sustainable way which does not adversely affect other infection prevention strategies within EH.

## Disclosure of interest

None declared

